# Rare Case of Spinal Neurosarcoidosis with Concomitant Epidural Lipomatosis

**DOI:** 10.1155/2021/5952724

**Published:** 2021-01-28

**Authors:** Nesreen Jaafar, Maria Khoueiry, Samia J. Khoury, Achraf Makki

**Affiliations:** ^1^Department of Neurology, American University of Beirut Medical Center, Beirut, Lebanon; ^2^Nehme and Therese Tohme Multiple Sclerosis Center, American University of Beirut Medical Center, Beirut, Lebanon

## Abstract

**Introduction:**

Spinal neurosarcoidosis is a rare disease that can manifest as myelopathy, radiculopathy, or cauda equine syndrome. Spinal epidural lipomatosis is also a rare condition resulting from overgrowth of epidural fat tissue causing compressive myelopathy. To our knowledge, there are no reports linking epidural lipomatosis and spinal neurosarcoidosis. *Case Report*. We describe a case of progressive myelitis in the presence of concomitant spinal neurosarcoidosis and epidural lipomatosis which was a challenging diagnosis with complete response to treatment after addressing both diseases. Both etiologies are inflammatory in nature and share similar expression of inflammatory factors such as TNF-*α* and IL-1*β*.

**Conclusion:**

The common inflammatory process involved in these two diseases might explain a pathophysiological interconnection between both diseases that may underlie their concomitant development in our patient. If these two diseases are interconnected, in their pathophysiological mechanism remains a hypothesis that will need further investigation.

## 1. Introduction

Sarcoidosis is a multisystem chronic disease that commonly presents with pulmonary manifestations [1]. Neurosarcoidosis is less common accounting for 5–16% of all sarcoidosis cases [2]. Spinal cord involvement is yet a very rare entity occurring at less than 1% of sarcoidosis patients [3], and because of its insidious, progressive, and nonspecific symptoms, it is usually a challenging diagnosis. Spinal neurosarcoidosis can present as transverse myelitis, myelopathy, cauda equine syndrome, radiculopathy, syringomyelia, or cord atrophy [4]. Myelitis from neurosarcoidosis was found to be an ultraextensive longitudinal myelitis, involving more than three consecutive vertebral body lengths [5]. Here, we present a case of spinal neurosarcoidosis initially presenting as epidural lipomatosis with cord compression. To our knowledge, there are no reports linking epidural lipomatosis and spinal neurosarcoidosis.

## 2. Case Presentation

A 43-year-old Lebanese man residing in Saudi Arabia presented to our medical center with bilateral lower extremity weakness, severe burning electric such as sensation in both lower extremities, and erectile dysfunction that was preceded by lower back pain. He is sexually active and overweight with no significant past medical history except for a treated sexually transmitted gonorrhea infection. He has no family history of relevant neurologic disease.

The symptoms progressed over one month with worsening neuropathic pain in both lower extremities, paresthesia starting at the umbilicus and radiating down to the medial thighs, inability to walk long distances due to lower extremities weakness and claudication, erectile dysfunction, dribbling and slow urine stream, and constipation. He underwent extensive workup in Saudi Arabia that showed a longitudinal transverse myelitis. Cerebrospinal fluid (CSF) studies showed mildly elevated protein (0.56 g/L), normal sugar (55 mg/dl), absent pleocytosis, and no oligoclonal bands. Magnetic resonance imaging (MRI) of the brain and cervical spine was negative, while MRI of the dorsal spine showed a high T2 signal extending from T4 to T9 vertebrae with enhancement at the T7-T8 level. According to his CSF and MRI results, he was diagnosed with inflammatory myelitis of unclear etiology. He received intravenous pulse steroids twice with no improvement, followed by a tapering dose of oral steroids without any improvement or stabilization of symptoms; so, he presented for further work up.

His physical exam was notable for a sensory level at T10 with allodynia in both lower extremities, very mild weakness in hip flexion on the right (4+/5), and an antalgic gait.

**Figure 1:**
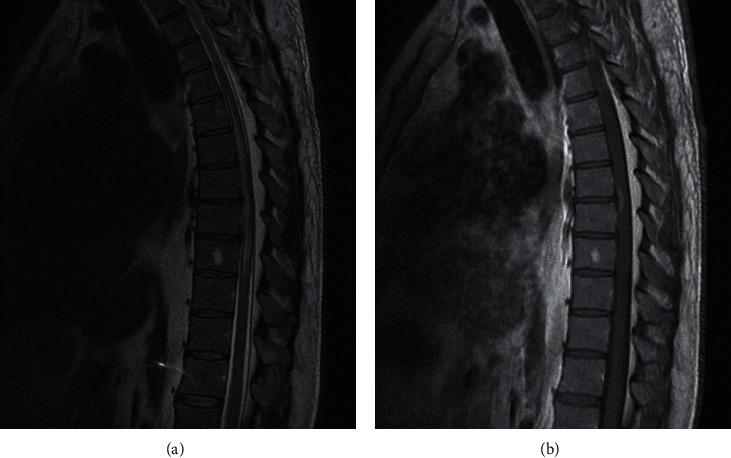
(a) Sagittal T2 MRI of dorsal spine showing high T2 signal with cord edema from T4 to T9 levels with thickened epidural fat tissue posteriorly. (b) Sagittal T1 MRI with gadolinium of dorsal spine showing an enhancing lesion at T7-T8 level.

**Figure 2:**
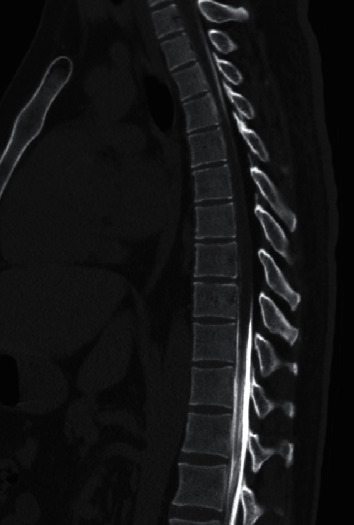
CT myelogram showing obstruction of CSF flow extending from T4 to T9 vertebrae.

MRI of the dorsal spine was repeated at our institution (Figure 1) showing the same longitudinally extensive dorsal lesion with diffuse cord edema and enhancement. The MRI also showed thickened epidural fat tissue encircling and effacing the CSF at these levels. This was followed by a computed tomography (CT) myelography (Figure 2) that showed near complete obstruction of CSF flow extending from T4 to T9 vertebrae. Autoimmune panel including antinuclear antibody, anti-Sjögren's syndrome-related antigen A autoantibodies, anti-Sjögren's syndrome-related antigen B autoantibodies, and antineutrophil cytoplasmic antibodies was negative. Infectious workup, including Brucella titers, human T-lymphotrophic virus serology, Toxocara antibodies, human immunodeficiency virus, and syphilis serology, was also negative. Antiaquaporin antibodies and antimyelin oligodendrocyte glycoprotein antibodies were negative too. Angiotensin converter enzyme, cobalamin, copper, and calcium levels were normal in blood.

**Figure 3:**
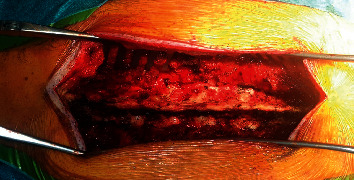
Intraoperative image showing the thickened epidural fat before excision.

With these MRI findings and negative autoimmune, infectious, demyelinating, and metabolic workup, the diagnosis of epidural lipomatosis causing compressive myelitis was raised, and neurosurgical evaluation recommended T4–T10 laminectomy for decompression and epidural fat removal (Figure 3).

After surgery, the patient reported a precipitous and significant improvement of his back pain, medial thighs burning sensation, and lower extremity weakness and claudication but persistent paresthesia, neuropathic pain, and erectile dysfunction.

**Figure 4:**
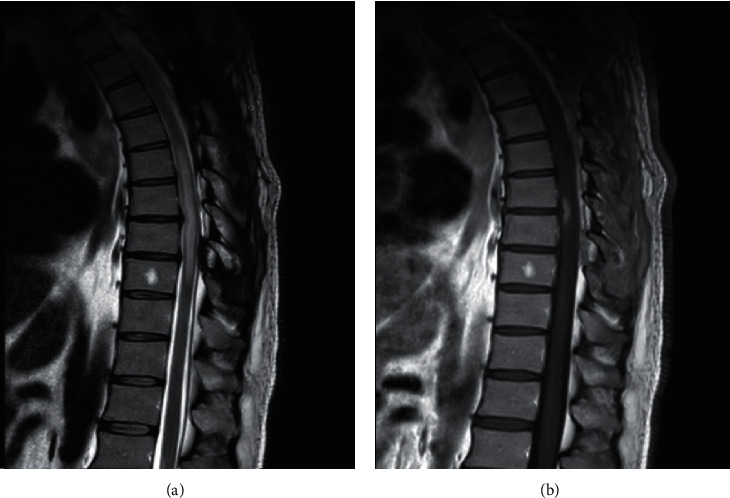
(a) Sagittal T2 MRI showing resolution of the epidural lipomatosis with resolution of the mass effect on the spinal cord and decrease in the extent of the lesion (from T6 to T9). (b) Sagittal T1 MRI with gadolinium showing mild decrease in the size of enhancing lesion.

**Figure 5:**
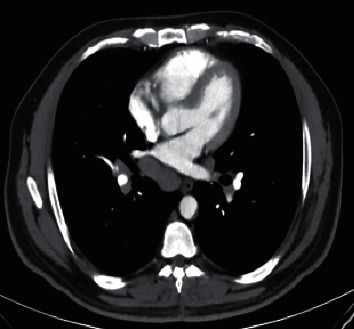
CT chest showing bilateral mediastinal and hilar adenopathy.

Four months later, during follow-up in the clinic, the patient reported persistence of his erectile dysfunction, with some remnant neuropathic pain, with normal motor function. A follow-up MRI spine (Figure 4) showed resolution of the epidural lipomatosis with resolution of the mass effect on the spinal cord and decrease in the extent of the lesion (from T6 to T9) with a decrease in the enhancement. However, mediastinal and hilar lymph nodes seemed to be enlarged on this MRI; so, a CT chest (Figure 5) was performed and confirmed this finding. This was followed by bronchoscopy and mediastinal lymph node biopsy that showed noncaseating granulomas. The diagnosis of pulmonary sarcoidosis was made and that raised the possibility of spinal neurosarcoidosis as the etiology of his residual neurologic symptoms and continued enhancement on the spinal imaging.

Since the patient has tried two courses of steroids in the early stages of his myelitis with no improvement, we started him on mycophenolate mofetil (Cellcept) 1000 mg twice daily as treatment for his sarcoidosis.

**Figure 6:**
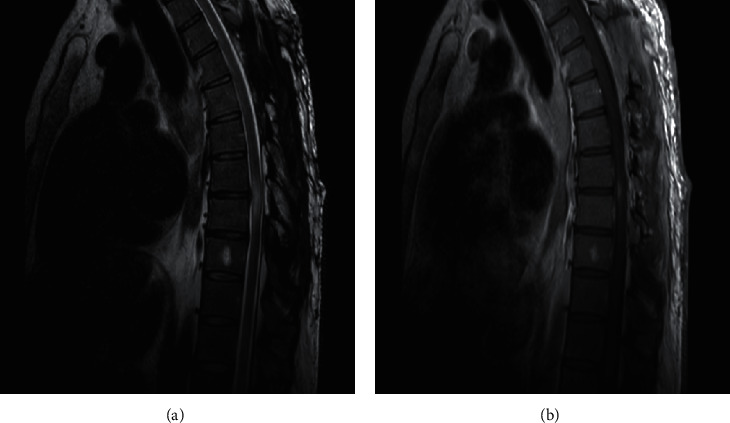
(a) Sagittal T2 MRI of whole spine after 2 months of treatment with mycophenolate mofetil showing decrease in T2 signal involving T7-T8. (b) Sagittal T1 MRI with gadolinium after 2 months of treatment showing further decrease in extent of enhancing lesion.

**Figure 7:**
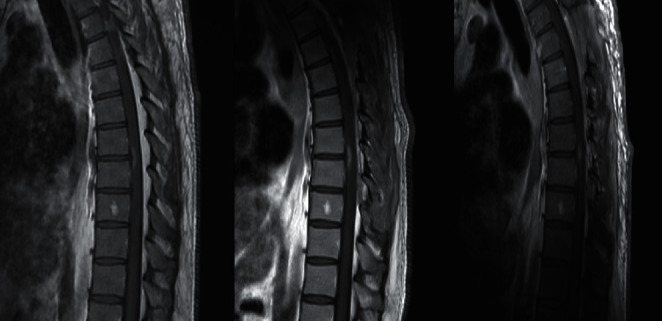
Sagittal T1 MRI with gadolinium showing the decrease in extent of enhancing lesion from initial MRI, after surgery, and after treatment.

Follow-up after two months of starting mycophenolate mofetil, he reported resolution of his erectile dysfunction, but persistent numbness and paresthesia mainly around the genital area. MRI of the dorsal spine showed decrease in the extent of the lesion to T7-T8 vertebrae with persistent enhancement anteriorly at this level (Figure 6). When comparing the enhancing lesion from initial MRI to the one after surgery and the one after treatment with mycophenolate mofetil, one can appreciate the marked decrease in the extent of enhancement (Figure 7). This decrease in enhancement is consistent with an inflammatory myelitis that can be a manifestation of neurosarcoidosis on top of spinal epidural lipomatosis. The gold standard diagnostic test to confirm spinal neurosarcoidosis, on the other hand, is a spinal cord biopsy, which we thought is too invasive to pursue in our patient. Given his improvement after treatment with mycophenolate mofetil and surgery, we considered a presumed diagnosis of spinal neurosarcoidosis with concomitant epidural lipomatosis that might have been exacerbated after steroid use.

## 3. Discussion

We report a case of spinal neurosarcoidosis with concomitant epidural lipomatosis presenting with longitudinally extensive myelitis that improved partially after surgical decompression and improved further after immunotherapy with mycophenolate mofetil. Due to the diversity in clinical presentations, rarity, and absence of the noninvasive confirmatory test, the diagnosis of spinal neurosarcoidosis is usually delayed especially when it is the first manifestation of sarcoidosis. This would be more challenging in a case such as ours in which there is concomitant pathology such as epidural lipomatosis that is also relatively rare and might explain this myelitis. Whether the resolution in the lesion was related to decrease in venous congestion after surgical repair of epidural lipomatosis only or is a combination of both responses to mycophenolate mofetil and surgery remains a possibility, given similar reported cases of epidural lipomatosis that improve after surgery [6].

Based on the clinical presentation and progression of symptoms, diagnostic considerations might include demyelinating diseases (e.g., multiple sclerosis and neuromyelitis optica), rheumatologic diseases (e.g., vasculitis, systemic lupus erythematosus, Sjogren syndrome, and Behcet's disease), infectious etiologies (e.g., brucellosis, Lyme disease, and tuberculosis), and neoplastic syndromes (e.g., leptomeningeal carcinomatosis) [7].

Spinal neurosarcoidosis can manifest as an acute/subacute process with cord lesions located in the dorsal more commonly than in the cervical spine [5]. Extraneural biopsy with the finding of noncaseating granulomas especially in the lungs is very helpful in the diagnosis [5]. Serum and CSF ACE levels are less sensitive and of limited value in the diagnosis of neurosarcoidosis, but elevated protein in the CSF is usually helpful [5]. The pathophysiology of sarcoidosis remains unknown; however, studies have shown elevated levels of tumor necrosis factor-*α*, interleukin-1*β*, and interleukin-6 secreted by alveolar macrophages in sarcoidosis patients when compared to alveolar macrophages of controls [8, 9]. Accordingly, steroids and cytotoxic drugs such as methotrexate, azathioprine, mycophenolate mofetil, and cyclophosphamide are used to alter the granulomatous inflammatory process and are the mainstay treatment in sarcoidosis [10]. Regarding the treatment of spinal neurosarcoidosis, it is largely based on experience in the absence of evidence based guidelines [4]. However, regardless of the treatment used, the recovery of the neurologic deficit depends on the severity of symptoms at initial presentation [11].

Spinal epidural lipomatosis is also a rare condition resulting from overgrowth of epidural fat tissue causing compressive myelopathy [12]. The main etiology of epidural lipomatosis is endogenous steroids overproduction or exogenous use of long-term steroids [12]. Nevertheless, idiopathic epidural lipomatosis and epidural lipomatosis in obese patients with an unclear etiology have been reported. A potential causal relationship between chronic inflammation and the development of epidural lipomatosis was suggested by studies that found higher expression levels of transcripts of TNF-*α* and interleukin-1*β* in adipocytes of patients with spinal epidural lipomatosis compared to healthy controls [13].

In our case, we describe a progressive myelitis in the presence of preexisting lipomatosis that worsened after the administration of exogenous steroids with compressive myelopathy and obstruction of CSF flow that is typical of epidural lipomatosis. However, the partial response after decompression by surgical removal of the lipomatosis and the presence of hilar and mediastinal enlarged lymph nodes with pathology showing noncaseating granuloma pointed to the diagnosis of neurosarcoidosis on top of the epidural lipomatosis. Furthermore, the resolution of symptoms and decrease in the extent of the enhancing lesion on MRI after starting mycophenolate mofetil can be explained by the concomitant presence of inflammatory myelitis in association with compressive myelitis.

Sarcoidosis (a chronic inflammatory disease) and epidural lipomatosis (a consequence of chronic inflammation) share similar expression of inflammatory factors such as TNF-*α* and IL-1*β* and could have interconnected pathophysiological mechanisms that may underlie the concomitant development of both diseases in our patient. However, this hypothetical relationship between these two entities will need further investigations.

## Data Availability

No data were used to support this study.
